# Cataract Surgery after Retinal Detachment Surgery with Arruga’s Sutures: Case Report

**DOI:** 10.4274/tjo.88864

**Published:** 2016-12-01

**Authors:** Erkan Ünsal, Kadir Eltutar, Osman Kızılay, Belma Karini

**Affiliations:** 1 İstanbul Training and Research Hospital, Ophthalmology Clinic, İstanbul, Turkey; 2 Zeynep Kamil Women’s and Children’s Hospital Training and Research Hospital, Ophthalmology Clinic, İstanbul, Turkey

**Keywords:** Retinal detachment, scleral buckling, Arruga’s suture

## Abstract

A 56-year old female patient presented to our clinic with a complaint of low vision in her right eye. Twenty-two years earlier she had undergone a scleral buckling operation in her right eye because of retinal detachment. She indicated that vision in her right eye was good after the surgery but had recently been gradually declining. Best-corrected vision acuity was counting fingers at 1 meter in the right eye and 8/10 in the left eye. Anterior segment examination revealed stage 3 nuclear cataract in the right eye. Examination of the right eye was blurred and revealed an area of chorioretinal atrophy posterior to the equator, approximately 3 disc diameters in the peripapillary zone and about 2 disc diameters in the nasal papilla zone. Anteriorly of the equator there was an area of chorioretinal atrophy as well as a narrow, sharply demarcated, shiny 360⁰ suture with high buckling pressure, situated intraretinally but extending into the vitreous in some places. The structure was thought to be made of polyethylene. Around the suture there were retinal atrophic changes. After detailed explanation of the possible surgical complications and after obtaining informed consent, the right eye cataract was removed by phacoemulsification and a foldable intraocular lens was placed into the capsule. During the operation, we worked under low fluid pressure and as atraumatically as possible due to the possibility of intraocular pressure changes and the risk of the suture causing retinal and blood vessel tears or passing completely into the eye and causing intravitreal hemorrhage. A month after an uncomplicated surgery, the posterior segment examination demonstrated a reattached retina and the patient’s best corrected visual acuity was 6/10.

## INTRODUCTION

Scleral buckling was commonly used in the past and is still utilized today in the treatment of retinal detachment. Although in recent years silicone-based structures have been used as encircling bands, Arruga sutures were also applied in the past.

In this report, we aimed to present a patient whose retinal detachment was treated with an encircling Arruga suture which years later caused intraocular invasion and cataract, necessitating cataract surgery.

## CASE REPORT

**Figure 1:**
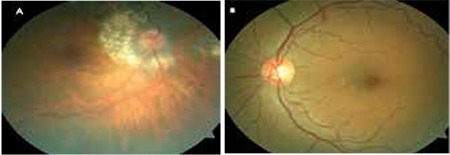
Posterior segment color photographs from the patient’s right (A) and left (B) eyes

**Figure 2:**
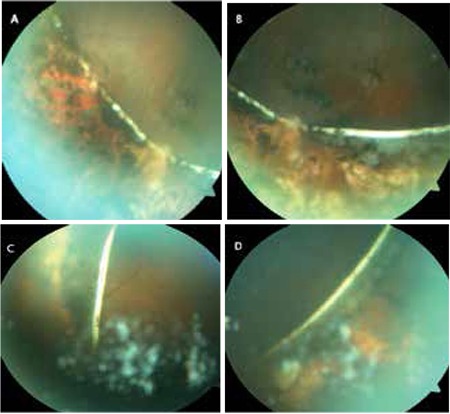
Appearance on fundus photography of the 360⁰ suture, located anterior to the equator, mainly situated intraretinally but showing some intrusion into the vitreous, believed to be polyethylene, inferotemporal (A), inferior (B), temporal (C) and inferonasal (D) views

**Figure 3:**
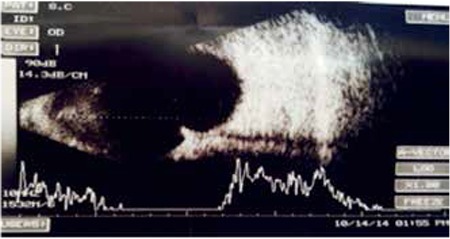
Ultrasonography of the inferotemporal quadrant showing hyperechogenic appearance of the Arruga suture invading the vitreous

A 56-year-old female patient presented to our clinic complaining of reduced vision in her right eye. She reported undergoing a scleral buckling procedure 22 years earlier due to retinal detachment in her right eye. She stated that her vision had been good after the procedure but had severely decreased recently. On ophthalmologic examination her vision was counting fingers from 1 meter in the right eye and 20/25 in the left eye. Anterior segment examination revealed stage 3 nuclear cataract in the right eye and nuclear sclerosis in the left eye. Intraocular pressure was within normal limits in both eyes. Fundus examination was natural in the left eye, while in the right eye a blurred region of chorioretinal atrophy was observed posterior of the equator and extending approximately 3 disc diameters in the peripapillary area and 2 disc diameters nasal of the papilla (Figure 1). Anteriorly of the equator there were areas of chorioretinal atrophy as well as a narrow, sharply demarcated, shiny 360⁰ suture with high buckling pressure (Figure 2). The suture was situated intraretinally but extended into the vitreous in some places, and was suspected to be made of polyethylene. Retinal atrophic changes were present surrounding the suture. Ultrasonography revealed a hyperechogenic structure which was believed to originate from Arruga suture that had invaded the vitreous (Figure 3).

The patient was given detailed information regarding possible complications and informed consent was obtained. Biometry measurements were acquired using partial coherence interferometry [intraocular lens (IOL) Master 500, Carl Zeiss Meditec, Germany] and the Sanders-Retzlaff-Kraff theoretic formula.

Anticipating that the patient may undergo other ocular surgeries in the future and because a 3-piece IOL would be more stable in such an event, a 5.5 mm optic diameter, 3-piece hydrophobic acrylic IOL was implanted in the capsule.

During the operation, we worked under low fluid pressure and as atraumatically as possible due to the possibility of intraocular pressure changes and the risk of the suture causing retinal and vascular tears or passing completely into the eye and causing intravitreal hemorrhage.

At 1 month after the uncomplicated procedure, the retina was reattached and the patient’s corrected visual acuity was 20/33. During the 3-month postoperative follow-up period, the IOL was centered in the capsule and the retina remained attached. The patient’s visual acuity also remained stable.

## DISCUSSION

All of the various techniques utilized in the management of retinal detachment aim to create an adhesion to prevent fluid exchange between the retinal pigment epithelium (RPE) and sensorial retina in the area surrounding the retinal tear, to thus enable RPE active transport and reabsorption of the subretinal fluid, to reduce the effects of vitreoretinal traction, and to prevent new tear formation.^1,2,3,4^

Schepens et al.^5^ introduced the scleral buckling procedure for the treatment of retinal detachment. In the procedure, binocular indirect ophthalmoscopy and scleral buckle are used to localize retinal tears. Following lamellar scleral dissection, diathermy is applied to the area of the inner lamella corresponding to the retinal tear. A nonabsorbable, 1.25 mm-wide polyethylene tube is then fixed to the dissected area with a polyethylene/silk suture. After the subretinal fluid drains, the tube is tightened to provide sufficient pressure and the flap is closed over the tube. This lengthy procedure is usually performed under general anesthesia.

The Arruga technique is a dated surgical technique which has become obsolete in the treatment of retinal detachment. This technique, performed under local anesthesia, was used to simplify the scleral buckling method and reduce operation time. After localizing the tear, full-thickness scleral diathermy is applied to the area. In order to make an indentation, a 3-0 nylon, Supramid or Mersilen suture is placed posterior to the equator, stabilized in the four quadrants, and later tightened to provide adequate pressure after the subretinal fluid has drained.

The phenomenon observed in these patients of postoperative intraocular intrusion of the suture has been termed the ‘clothesline phenomenon’.^6^ Intraocular invasion of the suture has been associated with various complications including recurrent retinal and vitreous hemorrhage, uveitis or recurrent retinal detachment.^6,7,8,9^

In our patient, it was clear that an Arruga suture which was placed 22 years earlier gradually invaded the sclera and choroid, eventually reaching the inner retinal layers and intravitreal space. However, despite the prolonged time since the surgery, our patient had not experienced any problems.

## CONCLUSION

Although the Arruga suture is no longer used in contemporary practice, we may still encounter complications related to this technique in patients who underwent the procedure in the past. With this report we wished to highlight the need to be prepared when faced with complications due to Arruga sutures in patients undergoing ocular procedures for other reasons.

### Ethics

Informed Consent: It was taken.

Peer-review: Externally and internally peer-reviewed.
